# Peripheral T-Cell Receptor β Repertoire Dynamics Correlate with Response to Anti-PD-L1 Therapy in Non-Small Cell Lung Cancer

**DOI:** 10.3390/cancers18142241

**Published:** 2026-07-13

**Authors:** Maria Goulielmaki, Sotirios P. Fortis, Anastasia Xagara, Panagiota Batsaki, Theodoros Loupis, Giannis Vatsellas, Dimitrios M. Vrachnos, Alexandra Voutsina, Filippos Koinis, Evangelia Chantzara, Katerina Oikonomaki, Stavroula Samara, Georgia Christopoulou, Pantelis Constantoulakis, Periklis Makrythanasis, Anna Koumarianou, Ioannis S. Pateras, Vasilis Georgoulias, Athanasios Kotsakis, Constantin N. Baxevanis, Angelos D. Gritzapis

**Affiliations:** 1 Cancer Immunology and Immunotherapy Center, Saint Savas Cancer Hospital, 11522 Athens, Greece; fortis@ciic.gr (S.P.F.); pmpatsaki@agsavvas-hosp.gr (P.B.); cnbaxevanis@biol.uoa.gr (C.N.B.); 2 Laboratory of Oncology, Faculty of Medicine, School of Health Sciences, University of Thessaly, 41110 Larissa, Greece; axagara@uth.gr (A.X.); thankotsakis@uth.gr (A.K.); 3 Hematology Research Lab, Clinical, Experimental and Translational Research Center, Biomedical Research Foundation Academy of Athens, 11527 Athens, Greece; tloupis@bioacademy.gr; 4 Greek Genome Center, Biomedical Research Foundation of the Academy of Athens, 11527 Athens, Greece; gvatsellas@bioacademy.gr (G.V.); pmakryth@med.uoa.gr (P.M.); 5 RNA Systems Biology Lab, Centre for Human Technologies, Fondazione Istituto Italiano di Tecnologia, Via Enrico Melen 83, 16152 Genoa, Italy; dimitriosmiltiadis.vrachnos@uniroma1.it; 6 Department of Biology and Biotechnologies, University of Rome Sapienza, Piazzale Aldo Moro 5, 00185 Rome, Italy; 7 Institute of Chemical Biology, National Hellenic Research Foundation, 11528 Athens, Greece; voutsina@eie.gr; 8 Department of Medical Oncology, University General Hospital of Larissa, 41110 Larissa, Greece; fkoinis@uth.gr (F.K.); valiaxantzara@gmail.com (E.C.); 9 Genotypos-Science Labs, 15343 Athens, Greece; koikonomaki@genotypos.gr (K.O.); rsamara@genotypos.gr (S.S.); gchristopoulou@genotypos.gr (G.C.); pconstantoulakis@genotypos.gr (P.C.); 10 Laboratory of Medical Genetics, Medical School, National and Kapodistrian University of Athens, 15343 Athens, Greece; 11 Department of Genetic Medicine and Development, Medical School, University of Geneva, Boulevard du Pont d’Arve 40, 1205 Geneva, Switzerland; 12 Hematology Oncology Unit, Fourth Department of Internal Medicine, Attikon University General Hospital, 12462 Athens, Greece; akoumari@yahoo.com; 13 2nd Department of Pathology, Medical School, Attikon University Hospital, National and Kapodistrian University of Athens, 12462 Athens, Greece; ipateras@med.uoa.gr; 14 First Department of Medical Oncology, Metropolitan General Hospital, 15562 Athens, Greece; georgsec@med.uoc.gr; 15 Flow Cytometry Unit, Department of Biology, National and Kapodistrian University of Athens, Panepistimiopolis, Ilissia, 15784 Athens, Greece

**Keywords:** non-small cell lung cancer, anti-PD-L1, durvalumab, peripheral TCRβ repertoire, biomarkers, TRBV genes, tumor mutational burden

## Abstract

**Background:** Immune checkpoint blockade (ICB) targeting the PD-1/PD-L1 axis has improved outcomes in non-small cell lung cancer (NSCLC), although reliable biomarkers for predicting benefit are still limited. **Methods:** In this exploratory study, we conducted a longitudinal analysis of the peripheral T-cell receptor (TCR) β repertoire in 28 patients with unresectable stage IIIb non-small cell lung cancer (NSCLC) who received anti-PD-L1 immunotherapy following chemoradiotherapy. Blood samples were collected at baseline and three months after treatment initiation. **Results:** At the cohort level, global TCR repertoire features such as diversity and richness did not change significantly over time. However, when looking at individual patients, more specific patterns became evident. Patients could be broadly separated based on changes in clonotype richness, with reductions generally accompanied by lower diversity and decreased convergent TCR frequency. We also observed differences in TRBV gene usage in relation to clinical outcome, with higher TRBV20-1 and lower TRBV28 frequencies tending to associate with improved survival and delayed disease progression. Interestingly, the disappearance of dominant clonotypes from the peripheral blood during treatment was linked to longer progression-free survival (PFS). In addition, patients with higher baseline blood plasma tumor mutational burden (bTMB) showed greater clonotype richness and were more likely to exhibit this clonotype loss. The combination of high bTMB and clonotype disappearance identified a subgroup of patients with particularly favorable outcomes. **Conclusions:** Overall, these results suggest that early responses to PD-L1 blockade may be reflected less in global TCR repertoire shifts, including clonality and diversity measures, and more in subtle changes in clonotype composition and dynamics, since the frequencies of certain TRBV genes and the disappearance of dominant clonotypes following ICB were associated with clinical outcomes integrating TCR profiling with bTMB and could therefore help refine patient stratification and improve the understanding of immune responses in NSCLC. Nevertheless, due to the small number of recruited patients, our study is exploratory and hypothesis-generating, and further validation in larger patient cohorts is warranted.

## Simple Summary

Despite the fact that the discovery of immune checkpoint inhibitors (ICIs) has revolutionized cancer treatment, reliable predictive biomarkers of response to ICIs in non-small cell lung cancer (NSCLC) patients are scarce. Herein, we analyzed the peripheral T-cell receptor (TCR) β repertoire in 28 patients with unresectable stage IIIb non-small cell lung cancer (NSCLC) who received anti-PD-L1 immunotherapy following chemoradiotherapy, and also combined it with the levels of blood plasma tumor mutational burden (bTMB). We found certain differences in TRBV gene usage, along with the disappearance of dominant clonotypes from the peripheral blood during treatment, that were linked to clinical outcomes. The combination of high bTMB and clonotype disappearance identified a subgroup of patients with particularly favorable outcomes, thus pointing to an integration of TCR and bTMB for therapeutic outcome prediction in NSCLC.

## 1. Introduction

Non-small cell lung cancer (NSCLC) remains a leading cause of cancer-related mortality worldwide, with a substantial proportion of patients presenting with unresectable locally advanced disease [1]. The introduction of immune checkpoint inhibitors (ICIs) targeting the programmed cell death-1 (PD-1) and programmed death-ligand 1 (PD-L1) pathway has improved the therapeutic outcomes of NSCLC by restoring antitumor T-cell activity and improving survival outcomes [2,3,4,5,6,7,8]. The therapeutic landscape for stage III NSCLC has changed drastically following the introduction of consolidation immunotherapy after definitive chemoradiotherapy (CRT), which has become the standard of care based on the PACIFIC trial [5]. Despite these advances, a substantial proportion of patients still experience disease recurrence or fail to derive durable clinical benefits, highlighting the need for reliable biomarkers for the prediction of treatment response [7,8,9]. Although PD-L1 expression is currently used in clinical practice, its predictive value remains limited, and the immunological mechanisms underlying response or resistance to this therapeutic scheme are poorly understood [10,11].

The T-cell receptor (TCR) repertoire reflects the adaptive immune system’s capacity to recognize and respond to tumor-associated antigens. Advances in high-throughput sequencing technologies have enabled comprehensive characterization of TCR diversity, clonotype composition, and clonal dynamics, providing insights into immune responses during cancer progression and treatment [12,13]. Previous studies have suggested that features of the TCR repertoire, including diversity and clonality, may correlate with clinical outcomes in patients receiving immune checkpoint blockade [14,15,16]. Since CRT induces systemic immune modulation and may reshape the T-cell repertoire before immunotherapy, characterization of peripheral T-cell receptor (TCR) dynamics has emerged as a promising strategy for identifying biomarkers of treatment efficacy. However, studies investigating TCR repertoire remodeling in patients with unresectable stage III NSCLC treated with definitive CRT and consolidation immunotherapy with anti-PD-L1 agents remain limited, and their clinical significance has yet to be fully established [17,18]. Beyond global measures of TCR repertoire diversity and clonality, the distribution of TCR beta variable (TRBV) gene families may provide additional biological insight into the antigen-specific immune responses. Preferential amplification of particular TRBV families can reflect selective recruitment and clonal expansion of T-cell populations that recognize tumor-associated or treatment-induced antigens, suggesting that TRBV usage may serve as a marker of ongoing antigen-specific immune responses [19,20]. Emerging evidence indicates that biased TRBV gene usage is associated with clinical outcomes and immunotherapeutic responses in NSCLC [14,21], supporting the hypothesis that specific TRBV families may have prognostic relevance rather than representing random variations within the TCR repertoire.

In addition to immunological parameters, tumor mutational burden (TMB) has emerged as a potential predictive biomarker for response to immunotherapy, as tumors with higher mutational loads are more likely to generate immunogenic neoantigens capable of eliciting T-cell responses [22,23]. The relationship between TMB and the TCR repertoire, however, has not been fully elucidated, and integrating these complementary biomarkers may provide a better understanding of tumor–immune interactions [24].

In this exploratory, hypothesis-generating study, we performed a longitudinal analysis of the peripheral TCRβ repertoire in patients with unresectable stage III NSCLC treated with anti-PD-L1 immunotherapy following chemoradiotherapy. We aimed to investigate how TCR repertoire characteristics evolve during treatment and to determine their association with clinical outcomes. In addition to evaluating global repertoire metrics, we investigated TRBV family usage alterations as a biologically motivated component of the adaptive immune response that may be associated with patient outcomes. Furthermore, we explored the interplay between TCR repertoire features and blood-based TMB to evaluate their combined potential as predictive biomarkers.

## 2. Materials and Methods

### 2.1. Patients and Samples

Twenty-eight patients with histologically confirmed stage IIIb NSCLC between February 2021 and January 2023 were selected for the present study. The clinicopathological characteristics are summarized in Table 1. Clinical data were collected upon diagnosis. The patients’ median age was 68.7 years old (range: 48.1–80.8), they were diagnosed with unresectable stage IIIb disease and received chemoradiotherapy (CRT) followed by durvalumab (PD-L1 inhibitor, Imfinzi, Astra Zeneca, London, UK), in line with the National Comprehensive Cancer Network (NCCN) guidelines; none of the patients had received any type of treatment before recruitment. The applied eligibility criteria consisted of age > 18 years, histologically confirmed diagnosis of NSCLC, unresectable clinical stage III, Eastern Cooperative Oncology Group (ECOG) Performance Status 0–2, ≥1% of tumor cells with positive PD-L1 expression in accordance with the European Medicines Agency (EMA) approved indication for consolidation durvalumab during the study period and disease control after completion of CRT, based on the RECIST 1.1. criteria.

Peripheral blood was collected directly into K2-ethylenediaminetetraacetic acid (EDTA) tubes (BD Biosciences, Heidelberg, Germany) by a physician and transferred to the laboratory for further processing. Blood collection took place just before immune checkpoint inhibition (ICI) initiation (baseline sample) and 3 months after the first ICI administration. The present study has been approved by local ethics and scientific committees, and it was performed according to the Declaration of Helsinki. All patients provided written informed consent concerning their participation in the study.

### 2.2. DNA Isolation

Total genomic DNA was extracted from 400 μL of peripheral blood using the MagMAX™ DNA Multi-Sample Ultra 2.0 Kit (Thermofisher Scientific, Waltham, MA, USA) according to the manufacturer’s instructions. Briefly, the blood samples were combined with Enhancer Solution and Proteinase K in a volume-dependent ratio, followed by incubation at 65 °C for 20 min to ensure protein digestion. Next, an appropriate volume of DNA-binding bead mix was added, and the samples were incubated with shaking at 800 rpm to facilitate DNA binding. Magnetic separation was used to pellet the beads, and supernatants were removed. Beads were subsequently washed sequentially with the appropriate wash solutions, followed by air-drying. DNA was eluted in 60 µL of pre-warmed Elution Solution and separated magnetically before transfer to a new vial. The purified DNA was quantified using the Qubit^TM^ dsDNA HS Assay Kit (Thermofisher, Waltham, MA, USA) on a Qubit Fluorometer 3.0 (Thermofisher, Waltham, MA, USA) and stored at −20 °C.

### 2.3. Next-Generation Sequencing of the TCR β Chain in the Purified DNA Samples

The Oncomine™ TCR Beta-SR Assay, DNA, was used to target and sequence the TCRβ gene locus, including the variable (V), diversity (D), joining (J), and constant regions. The complementarity-determining region 3 (CDR3) straddles the V (D) J junction and is the primary site of antigen contact. The sequencing and identification of unique TCR clonotypes in the patients’ samples can help to accurately identify and measure the clonal expansion of T cells in the peripheral blood. A total DNA input of 1 μg was used for library construction. For the target amplification reactions and the successive library preparation, the Ion AmpliSeq^TM^ Library Kit Plus (Ion Torrent^TM^, Thermofisher, Waltham, MA, USA) and the Ion Torrent^TM^ Dual Barcode Kit 1–96 (Ion Torrent^TM^, Thermofisher, Waltham, MA, USA) were used, respectively. The amplified libraries were then purified using the Agencourt^TM^ AMPureTM XP Reagent (BeckmanCoulter Life Sciences, Brea, CA, USA) and were individually quantified by qPCR using the Ion Library TaqMan^TM^ Quantitation Kit (Ion TorrentTM, Thermofisher, Waltham, MA, USA) on the Quantstudio 5.0 (Thermofisher, Waltham, MA, USA). The quantified libraries were diluted to 25 pM of individual concentration and pooled. After template preparation, the libraries were loaded on an Ion 530^TM^ Chip using the Ion Chef^TM^ System, and finally, sequencing was achieved on the Ion GeneStudio^TM^ S5 System (all from Ion Torrent^TM^, Thermofisher, Waltham, MA, USA) (single-end sequencing, 80 bp average nucleotide length).

### 2.4. TCR Sequencing Data Analysis

Immune repertoire analysis was performed using the Ion Reporter™ Software 5.16 with the Oncomine TCR Beta-SR—w1.2—DNA—Single Sample workflow (both from Ion Torrent; Thermo Fisher Scientific, Inc.). The software reported the total detected variable (V)—joining (J) region rearrangements and provided the number of productive and unproductive reads for each sample. The repertoire metrics (clonotype richness, Shannon diversity index, Gini diversity index, evenness, and convergent TCR frequency, CDR3 length) were generated using the standard Ion Reporter v5.16 Oncomine TCR Beta-SR workflow. Clonotype richness represented the total number of TCR clonotypes. TCR convergence was defined as the aggregate clonotype frequency with a shared variable gene and CDR3 amino acid sequence. The Gini and Shannon diversity indexes are interrelated metrics of the TCR repertoire; the Gini index records clonality inequality, with high values indicating strong clonal expansion or a highly skewed repertoire, while the Shannon index measures diversity by combining the number of unique clones (richness) and their relative frequencies (evenness). V-gene usage values were also obtained and used for further statistical analysis. The identified clonotypes per patient were put in rank order based on their frequencies. For each clonotype, the identified V and J genes were listed, as well as the amino acid (AA) sequence of the CDR3 locus (CDR3 AA).

### 2.5. Cell-Free DNA (cfDNA) Extraction

Whole blood was collected in Streck Cell-Free DNA (cfDNA) BCT tubes (Streck, La Vista, NE), and plasma was collected by double centrifugation within 4 to 48 h according to the manufacturer’s guidelines. Plasma samples were stored at −80 °C until cfDNA extraction. cfDNA was isolated from a minimum of 4 mL of plasma using the QIAamp Circulating Nucleic Acid Kit (Qiagen, Hilden, Germany), according to the manufacturer’s guidelines. Extracted cfDNA was quantified using the Qubit dsDNA High-Sensitivity Assay Kit (Thermo Fisher Scientific, Waltham, MA, USA). The isolated cfDNA was stored at −80 °C.

### 2.6. Library Construction and Next-Generation Sequencing of Circulating Tumor DNA (ctDNA)

Circulating tumor DNA (ctDNA) libraries were prepared from plasma-extracted cfDNA (minimum input: 30 ng) using the TruSight™ Oncology 500 (TSO500) ctDNA v1 kit (Illumina Inc, San Diego, CA, USA) protocol, according to the manufacturer’s protocol. In brief, cfDNA underwent end repair and A-tailing, followed by unique molecular identifier (UMI) ligation and PCR indexing in batches of 24 samples. Target enrichment was achieved through an initial overnight hybridization of the libraries with biotinylated probes. The next day, the first capture of targeted sequences was performed using streptavidin magnetic beads, followed by a second hybridization and a subsequent stringency capture to maximize library specificity. Libraries were quantified, normalized, and pooled for sequencing. Sequencing was performed on a NovaSeq 6000 instrument using an S4 flow cell (2 × 150 bp). High-depth sequencing was performed with a minimum data output of 100 Gb per sample to ensure the detection of low-frequency variants.

### 2.7. Bioinformatics Pipeline for ctDNA Data Analysis

Raw sequencing data were processed at the Biomedical Research Foundation of the Academy of Athens (BRFAA). Following demultiplexing and quality control, reads underwent adapter trimming and removal of low-quality bases before alignment to the human reference genome (hg19). High-quality consensus reads were generated by leveraging UMI technology to suppress stochastic sequencing errors and enhance the detection of low-frequency variants. Somatic variant calling was performed in accordance with the GATK (Genome Analysis Toolkit, v4.5.0.0) Best Practices [25].

The blood plasma Tumor Mutational Burden (bTMB) was computed over protein-coding regions with coverage ≥ 1000× following the criteria established by the Illumina TruSight Oncology 500 (TSO500) protocols for ctDNA. To ensure an accurate estimation and minimize biological noise, the following filtration layers were applied [26,27]: (a) Variants with a population allele count ≥ 10 in the gnomAD v2.1.1 database were excluded as germ-line variants. (b) Variants in the commonly mutated genes in Clonal Hematopoiesis (CH), DNMT3A, TET2, PPM1D, and ASXL1 were removed; additionally, for variants with a variant allele count (VAC > 50), fragment size analysis was used to distinguish CH-derived fragments (typically longer) from tumor-derived cfDNA. (c) Variants with count ≥ 50 in the COSMIC database were considered as tumor driver variants and excluded from the bTMB calculations in order to reduce bias due to targeted enrichment of the panel of genes. The final bTMB score was calculated using only nonsynonymous variants with a frequency ≥0.5\%bTMB = Eligible Variants/Effective Panel Size (Mbp).

### 2.8. Statistical Analysis

Data plotting and statistical analyses were performed using GraphPad Prism 9.4.1 for Windows (GraphPad Software, Inc., San Diego, CA, USA). The non-parametric Wilcoxon test was used for the identification of statistically significant differences in the patients’ TCR repertoire metrics between the two examined timepoints, and the Tukey whisker plots present the corresponding medians ± standard deviations (SD). Statistically significant differences in V-gene usage among patients and between baseline and first evaluation (1stE) were identified by 2-way ANOVA, Fisher’s LSD test for multiple comparisons, and the data are plotted as mean ± SD; however, multiple hypothesis testing using Benjamini–Hochberg FDR was also performed, and the adjusted *p*-values are also reported. For survival analysis, the Kaplan–Meier curves for overall survival (OS) and progression-free survival (PFS) were plotted, and the log-rank and the Gehan–Breslow tests were used for the estimation of the statistical significance. Cox regression analysis was used for univariate and multivariate analysis for the evaluation of the prognostic significance of TCR clonotype disappearance in association with selected clinicopathological characteristics as covariates. For the analysis of bTMB, estimated through sequencing of ctDNA, the non-parametric Mann–Whitney test was used to assess differences between patients with high versus low baseline clonality, while the Wilcoxon test was applied to compare clonality within patient groups between baseline and 1stE. A threshold of 10 mutations per megabase (mut/Mb) was used to define high versus low bTMB. To examine the association between bTMB and disappeared clonotypes, patient counts within each group were percentage-transformed and analyzed using Fisher’s exact test for contingency analysis. A *p*-value < 0.05 was considered statistically significant.

## 3. Results

### 3.1. The Effects of Anti-PD-L1 Therapy Post Chemoradiotherapy on TCR Repertoire Characteristics of Patients with NSCLC

**Figure 1:**
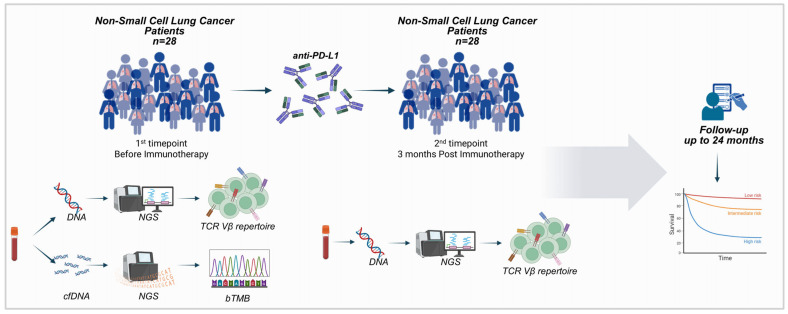
Schematic representation of the study’s research design. [Created in BioRender. Fortis, S. (2026) https://BioRender.com/vfqu5oi, accessed on 9 July 2026]. Agreement license QA29WQ2IL7. NGS, next-generation sequencing; cfDNA, cell-free DNA; bTMB, blood tumor mutational burden; TCR Vβ, TCR variable beta chain.

Twenty-eight patients with unresectable, stage IIIb NSCLC were enrolled in the present study (Table 1). All patients had received various cycles of chemoradiotherapy prior to the time of first sample collection (baseline timepoint). A second blood collection was scheduled at 3 months after ICI initiation (1st evaluation timepoint; 1stE). The median age of the patients was 68.7 years (range: 48.1–80.8). The patients were followed up for 2 years after the diagnosis for correlations between clinical outcomes and TCR repertoire findings. The study design is presented in Figure 1.

**Figure 2:**
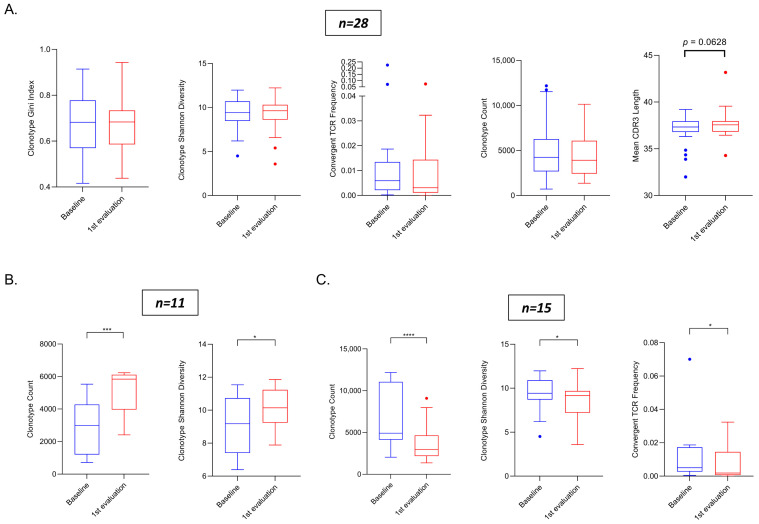
TCRβ repertoire metrics in the peripheral blood of patients with NSCLC. The comparative analysis was performed on NGS data from whole blood samples from patients with unresectable stage III NSCLC at baseline and 3 months after anti-PD-L1 treatment initiation (1st evaluation; 1stE). Potential therapy-induced alterations in T-cell complexity were analyzed in all patients (*n* = 28) with respect to (**A**) Gini index, indicating the level of clonal expansion; Shannon diversity (entropy) index, expressing patient clonotype diversity; convergent TCR frequency, estimated as the aggregate clonotype frequency with shared variable gene and CDR3 amino acid sequence but different nucleotide space; and TCR clonotype counts and CDR3 aminoacid sequence length. Based on clonotype count at 1stE compared to baseline, patients could be categorized into two groups: (**B**) increased clonotype count, also showing increased Shannon diversity index (*n* = 11), and (**C**) decreased clonotype count, also characterized by decreased Shannon diversity index and convergent TCR frequency (*n* = 15). Regarding (**B**,**C**), patient number (*n* = 26) differs from the total (*n* = 28), since two patients did not have altered clonotype counts at 1stE. Data are presented as the median value of patients ± SD. The asterisks designate a statistically significant difference indicated by a *p*-value below 0.05 (* *p* < 0.05; *** *p* < 0.001; **** *p* < 0.0001). TCRβ, T-cell receptor chain β; NSCLC, non-small cell lung cancer; NGS, next-generation sequencing; SD, standard deviation.

To explore the effect of anti-PD-L1 treatment on the T-cell receptor (TCR) Vβ repertoire, T-cell complexity was analyzed both at baseline and at 1stE. On average, 393.154 sequence reads were obtained, which were mapped to the V and J segments, and could identify unique TCR Vβ clonotypes. Analysis of the T-cell receptor (TCR) repertoire characteristics—including diversity, evenness, richness, and convergent TCR frequency—in NSCLC patients showed no significant differences at the 1stE evaluation timepoint compared with baseline. However, there was a trend toward longer mean CDR3 sequence lengths at the later timepoint (Figure 2A). Although no statistically significant differences in TCR repertoire metrics were observed between the two selected timepoints, patients could clearly be separated into two groups based on the clonotype richness of their TCR repertoire; 11 patients with increased clonotype counts (*p* = 0.001) also displayed enhanced diversity (*p* = 0.0316) at 1stE, reflecting a highly complex and heterogenous repertoire of TCR clonotypes (Figure 2B). On the contrary, the decreased clonotype richness (*p* < 0.0001) noted in 15 patients at 1stE was accompanied by both a statistically significant decrease in repertoire diversity (*p* = 0.0353) and frequency of convergent TCRs (*p* = 0.0256) (Figure 2C), pointing out to a narrowed, less diverse immune repertoire.

### 3.2. Specific TRBV Genes Associate with Improved Clinical Responses to Anti-PD-L1-Based Immunotherapy in NSCLC Patients

**Figure 3:**
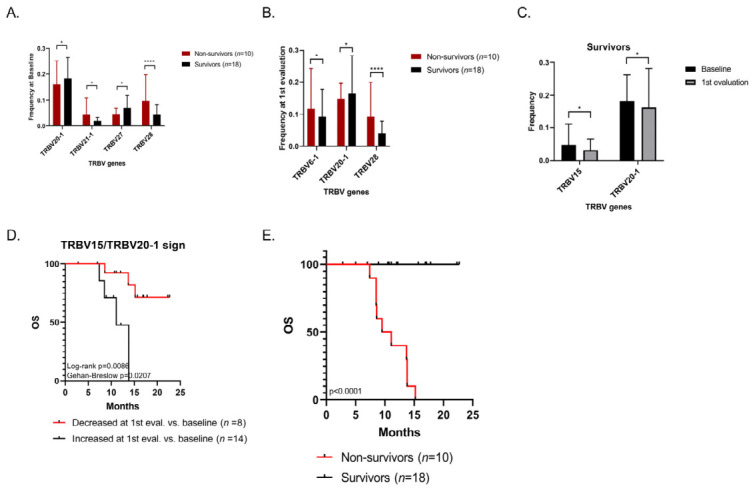
Deviations in TRBV-gene frequencies in NSCLC patients based on survival. TRBV genes with statistically significantly different frequencies in survivors (alive at last follow-up) vs. non-survivors (dead at last follow-up) at baseline (**A**) and 1st evaluation (**B**,**C**). TRBV genes with statistically significantly different frequencies at baseline compared to 1stE in survivors. Data are plotted as the mean value ± SD. The asterisks indicate a statistically significant difference, indicated by a *p*-value below 0.05 (* *p* < 0.05; **** *p* < 0.0001). (**D**) Survival analysis based on the combined frequency levels of TRBV15 and TRBV20-1. The Kaplan–Meier survival curves were designed based on the increased or decreased frequency of the combination of TRBV15 and TRBV20-1 at 1stE compared to baseline. Patients with decreased frequency of both TRBV genes at 1stE (red lines; decreased compared to baseline) had statistically significantly better overall survival (OS) as compared to patients with increased frequency of both TRBV genes at 1stE (black lines; increased compared to baseline). (**E**) A 2-year OS analysis in survivors vs. non-survivors at last follow-up. The Kaplan–Meier survival curves were designed based on the dichotomization of patients into survivors and non-survivors at their last follow-up. Both the log-rank and the Gehan–Breslow methods were used for the identification of statistically significant differences between the survival curves.

The correlation between TRBV-gene occurrences and survival revealed certain differences in the clonal frequencies (CFs) of survivors compared to non-survivors at the last follow-up, both at baseline and at 1stE (Figure 3A,B). Comparison of the total TRBV-gene frequencies at the two selected timepoints between survivors and non-survivors underscored four TRBV genes with differential expression at baseline (Figure 3A) and three TRBV genes with significantly different CFs at 1stE (Figure 3B). TRBV20-1 was found at higher frequencies and TRBV28 at lower frequencies in survivors at both timepoints (Figure 3A,B); regarding the other TRBV-genes, survivors had increased frequencies of TRBV27 and decreased frequencies of TRBV21-1 compared to non-survivors at baseline (Figure 3A), while at 1stE, decreased incidence of TRBV6-1 was identified among the group of survivors (Figure 3B). However, it should be mentioned that because this study is exploratory and based on a relatively small cohort, we used Fisher’s LSD test for multiple comparison ANOVA, since multiple hypothesis testing using Benjamini–Hochberg FDR or other correction tests resulted in non-significant results, with the exception of TRBV28 frequency, which remained statistically significant in both timepoints (adjusted *p* < 0.0001). Patients who were alive at their last follow-up showed alterations in the frequencies of specific TRBV genes in their blood at 1stE compared to baseline (Figure 3C). Within this group of survivors, a statistically significant decline in the frequencies of TRBV15 and TRBV20-1 was noticed at 1stE compared to baseline (Figure 3C). Notably, the concomitant downregulation in the frequencies of TRBV15 and TRBV20-1 at 1stE compared to baseline was associated with statistically significant improved OS in contrast to upregulated frequencies of both TRBV genes (Figure 3D). The Kaplan–Meier survival curve for OS is graphed in Figure 3E.

We also investigated whether baseline and 1stE TCR repertoire characteristics were associated with OS. Comparisons of major repertoire metrics, including clonotype richness and diversity, revealed no statistically significant differences between patients with favorable and unfavorable clinical outcomes (all *p* > 0.05, Figure S1).

**Figure 4:**
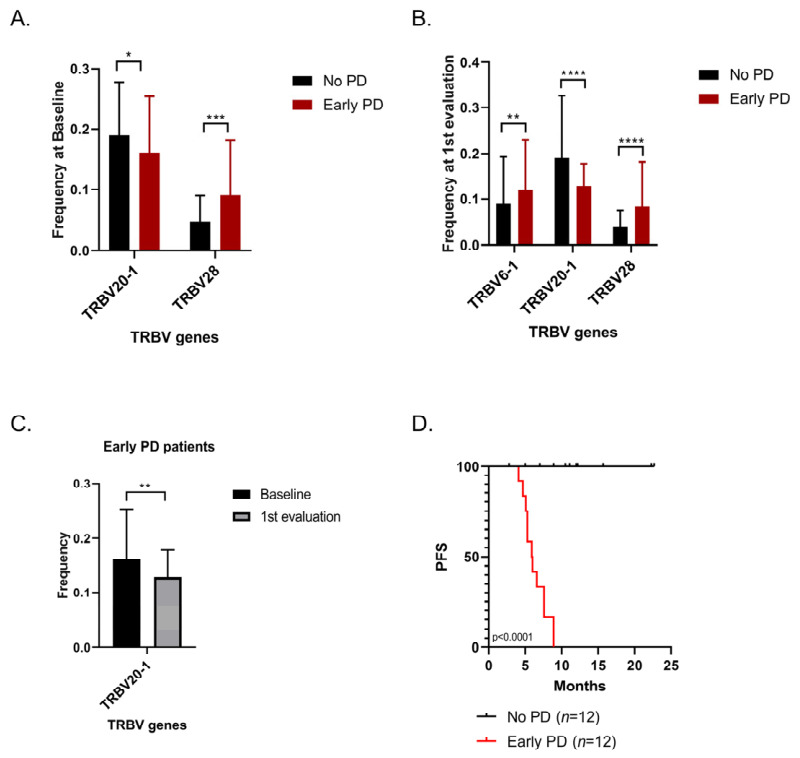
Differences in TRBV-gene frequencies in NSCLC patients based on the presence or absence of progressive disease. (**A**) TRBV genes with statistically significant differences in frequencies in patients without progressive disease (no PD) vs. patients with early progressive disease (early PD) at baseline. (**B**) TRBV genes with statistically significant differences in frequencies in patients without progressive disease (no PD) vs. patients with early progressive disease (early PD) at 1st evaluation. (**C**) TRBV genes with statistically significant differences in frequencies at baseline compared to 1st evaluation (1stE) in early PD patients. Data are plotted as the mean value ± SD. The asterisks indicate a statistically significant difference indicated by a *p*-value below 0.05 (* *p* < 0.05; ** *p* < 0.01; *** *p* < 0.001; **** *p* < 0.0001). (**D**) A 2-year progression-free survival (PFS) analysis in no PD vs. early PD patients. The Kaplan–Meier survival curves were designed based on the dichotomization of patients into no PD and early PD by the end of the study. Both the log-rank and the Gehan–Breslow methods were used for the identification of statistically significant differences between the survival curves.

In similar lines, patients with no progressive disease (PD) were characterized by significant differences in the CFs of certain TRBV genes compared to patients with documented early PD events (early PD was considered when it occurred at 10 months after the initiation of immunotherapy or earlier). At baseline, patients with no PD had increased frequencies of TRBV20-1 and decreased frequencies of TRBV28 compared to early PD patients (Figure 4A). The increased frequencies of TRBV20-1 and decreased frequencies of TRBV28 in no PD patients vs. early PD patients were maintained at 1st evaluation, alongside decreased rates of TRBV6-1 (Figure 4B). Notably, TRBV20-1 frequencies were significantly decreased in early PD patients at 1stE (Figure 4C). As expected, early PD patients had an unfavorable prognosis with the shortest PFS compared to no PD patients (Figure 4D). Again, multiple hypothesis testing using Benjamini–Hochberg FDR resulted in only TRBV28 frequency remaining statistically significant for early PD vs. no PD at baseline (adjusted *p* = 0.0068), while only TRBV20-1 and TRBV28 had a statistically significant *p*-value at 1st evaluation (TRBV20-1; adjusted *p* < 0.0001, TRBV28; adjusted *p* = 0.0022).

### 3.3. The Disappearance of TCR Clonotypes from the Peripheral Blood of NSCLC Patients After Immunotherapy Correlates with Favorable Clinical Outcomes

**Figure 5:**
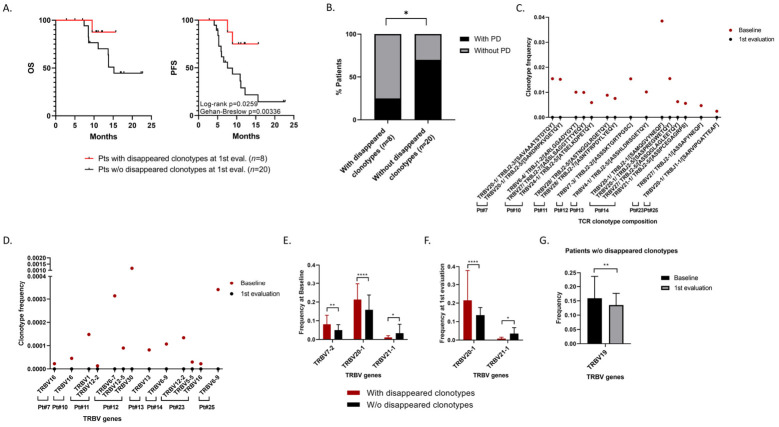
Association between baseline top 10 most frequent TCRVβ (10mfTCRVβ) clonotypes that disappeared from the peripheral blood of NSCLC patients after immunotherapy, with clinical outcomes. (**A**) Survival analysis based on the presence or absence of disappeared clonotypes at 1st evaluation. The Kaplan–Meier survival curves were designed based on the presence or absence of 10mfTCRVβ disappeared clonotypes in the peripheral blood of patients at 1st evaluation. Patients with 10mfTCRVβ disappeared clonotypes at 1st evaluation (1stE) (red lines) had a favorable, though not significant, 2-year overall survival (OS) and a statistically significant better 2-year progression-free survival (PFS) as compared to patients without 10mfTCRVβ disappeared clonotypes at 1stE (black lines). (**B**) Analysis of the contingency between the disappearance of TCRβ clonotypes from the peripheral blood of NSCLC patients after immunotherapy and the presence or absence of progressive disease (PD). The asterisks designate a statistically significant difference indicated by a *p*-value below 0.05 (* *p* < 0.05). (**C**) Frequencies and TRBV-gene, TCR beta joining (TRBJ)-gene, and CDR3 composition of the 10mfTCRVβ disappeared clonotypes in NSCLC patients. The dot plot depicts the frequency alterations in the corresponding clonotypes per patient at baseline. The specific TRBV- and TRBJ-genes and the amino acid sequence of each clonotype are also presented. (**D**) Graphical representation of all TRBV genes that disappeared from the peripheral blood of patients at 1st evaluation. The dot plot presents specific TRBV genes that were present in the peripheral blood of the enlisted patients at baseline and were undetectable at 1st evaluation. TRBV genes with statistically significantly different frequencies in patients with 10mfTCRVβ disappeared clonotypes at baseline (**E**) and 1stE (**F**,**G**). TRBV genes with statistically significantly different frequencies at baseline compared to 1stE in patients with 10mfTCRVβ disappeared clonotypes. Data are plotted as the mean value ± SD. The asterisks indicate a statistically significant difference indicated by a *p*-value below 0.05 (* *p* < 0.05; ** *p*< 0.01; **** *p* < 0.0001).

Apart from the overall alterations in V-gene frequencies, we also investigated individual changes in CFs among the top 10 most frequent TCR Vβ (10mfTCRVβ) clonotypes at 1stE for each patient separately (Figure S2). Interestingly, a subset of eight patients, namely patients #7, #10, #11, #12, #13, #14, #23, and #25, had different numbers of various 10mfTCRVβ clonotypes that disappeared from their peripheral blood at 1stE. Although OS did not differ in a statistically significant manner, those patients with disappeared 10mfTCRVβ clonotypes had statistically significantly improved 2-year PFS compared with patients with no disappeared 10mfTCRVβ clonotypes (Figure 5A). Contingency analysis revealed that the frequency of progressive disease differed significantly based on TCR clonotype disappearance (*p* < 0.05). Patients with TCR clonotype disappearance exhibited a substantially lower proportion of progressive disease (25%) than patients without clonotype disappearance (70%), whereas the proportion of patients without progressive disease was correspondingly higher (75% vs. 30%), supporting an association between TCR clonotype disappearance and improved clinical outcome (Figure 5B). The number, CFs at baseline, V-J recombination, and CDR3 sequences of the disappeared clonotypes are depicted in Figure 5C. Notably, each patient’s peripheral blood sample at 1stE was characterized by a different number of disappeared 10mfTCRVβ clonotypes; four patients had 1, two patients had 2, one patient had 3, and one patient had 4 disappeared 10mfTCRVβ clonotypes. Out of the total 15 10mfTCRVβ clonotypes that disappeared, five consisted of TRBV20-1, three consisted of TRBV27, and two consisted of TRBV28, and their CFs at baseline ranged from 0.002429 to 0.03849 (mean 0.01143 ± 0.008617). Subsequently, looking into the whole TCRβ repertoire of each patient, the baseline frequencies of certain TRBV genes were eliminated at 1stE (Figure 5D). TRBV16 was a commonly disappeared V-gene in three patients, followed by TRBV12-2 and TRBV6-9, each being absent from the peripheral blood of two patients. Interestingly, none of the latter TRBV genes were constituents of the baseline 10mfTCRVβ clonotypes that disappeared at 1stE. The analysis of the TCRβ repertoire of the total patient cohort showed that patients with 10mfTCRVβ clonotypes at baseline that disappeared at 1stE, as opposed to patients whose 10mfTCRVβ clonotypes did not disappear at 1stE, had increased frequencies of TRBV20-1 and TRBV7-2 and decreased frequencies of TRBV21-1 at baseline (Figure 5E); these differences in TRBV20-1, TRBV7-2, and TRBV21-1 between the two patient groups were retained at 1stE (Figure 5F). Regarding the group of patients without disappeared clonotypes, anti-PD-L1 treatment downscaled the frequencies of TRBV19 in their peripheral blood (Figure 5G). Multiple hypothesis testing using Benjamini–Hochberg FDR resulted in only alterations in TRBV20-1 frequency between patients with and those without disappeared TCR clonotypes, maintaining a discovery with adjusted *p*-values below 0.0001 for both timepoints.

To investigate the prognostic significance of TCR clonotype disappearance from the peripheral blood, we conducted univariate and multivariate analyses, using established clinicopathological factors (i.e., age, smoking status, ECOG, histological type) and the disappearance of TCR clonotypes as covariates and 2-year PFS as endpoint (Table 2). In the multivariate analysis, the prognostic significance of the five covariates (TCR clonotype disappearance, age, smoking status, ECOG, and histological type) was analyzed. Age was significantly associated with increased hazard of progressive disease. Each one-year increase in age was associated with an 8.0% increase in hazard (HR = 1.08, 95% CI: 1.003–1.171). Squamous histology was also associated with a significantly higher hazard compared with adenocarcinoma (HR = 3.43, 95% CI: 1.004–14.03), corresponding to approximately a 3.4-fold higher risk. Although TCR clonotype disappearance did not reach statistical significance (*p* = 0.1661), in the univariate analysis, the absence of TCR clonotype disappearance was significantly associated with a higher hazard of disease progression; patients without TCR clonotype disappearance had a 4.62-fold increased hazard compared with those with TCR clonotype disappearance (HR = 4.62, 95% CI: 1.28–29.56; *p* = 0.0441). Although statistically significant, the wide confidence interval indicates limited precision of the effect estimate. Increasing age was also significantly associated with shorter PFS (HR = 1.08, 95% CI: 1.00–1.17; *p* = 0.0488). Histological type showed a trend toward significance, with squamous histology associated with a higher hazard than adenocarcinoma (HR = 2.58, 95% CI: 0.92–8.33; *p* = 0.0849). The fact that other established prognostic factors, including ECOG performance status and smoking status, did not prove strong prognosticators of 2-year PFS could be associated with the small patient cohort size and would require further investigation.

### 3.4. Peripheral Blood TMB Combined with the TCRβ Repertoire Correlates with Response to ICB in NSCLC

**Figure 6:**
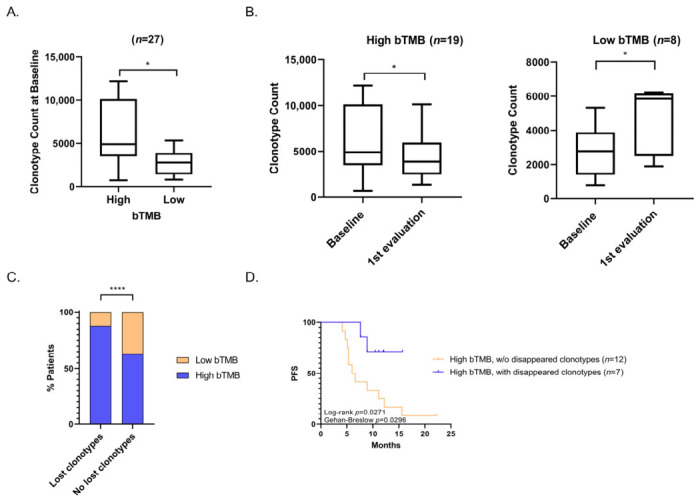
Combined assessment of peripheral blood plasma tumor mutational burden (bTMB) and TCRβ repertoire in NSCLC patients. Whisker plots represent differences in TCRβ clonotype count at baseline between patients with high vs. patients with low bTMB (**A**), and at baseline vs. 1st evaluation (1stE) in patients with high bTMB and low bTMB (**B**). Data are plotted as the median value ± SD. (**C**) Analysis of the contingency between bTMB levels and baseline top 10 most frequent TCRVβ (10mfTCRVβ) clonotypes that disappeared from the peripheral blood of NSCLC patients after immunotherapy. The graphical bars show percentages of patients with 10mfTCRVβ disappeared clonotypes vs. without disappeared clonotypes at 1stE in association with high vs. low bTMB levels. The asterisks designate a statistically significant difference indicated by a *p*-value below 0.05 (* *p* < 0.05; **** *p* < 0.0001). (**D**) 2-year progression-free survival (PFS) analysis based on the combined bTMB levels and the presence or absence of 10mfTCRVβ disappeared clonotypes at 1st evaluation. The Kaplan–Meier survival curves were designed based on the dichotomization of patients into high bTMB and presence of 10mfTCRVβ disappeared clonotypes (purple line) vs. high bTMB and absence of 10mfTCRVβ disappeared clonotypes (orange line) at 1stE.

We next investigated the potential association between baseline bTMB levels, estimated through sequencing of ctDNA, and TCR repertoire characteristics. Sequencing data were available for 27 patients, while one patient had insufficient sample material for analysis. Patient samples were grouped as high vs. low bTMB based on a cut-off of 10 mut/Mb. As shown in Figure 6A, patients with high bTMB were defined by higher TCRβ clonotype richness compared to patients with low bTMB at baseline (*p* = 0.0112). Subgrouping of patients based on their baseline bTMB levels revealed a statistically significant decrease in clonotype richness at 1stE in patients with high bTMB (*p* = 0.0150), while a significant increase in TCRβ clonotype counts at 1stE was found for patients with low bTMB (*p* = 0.0341) (Figure 6B). Among the eight patients with disappeared (10mfTCRVβ) clonotypes from the peripheral blood at 1stE (see Figure 5A), seven patients (87.5%) had high bTMB (Figure 6C), whereas the incidence of patients without disappeared 10mfTCRVβ clonotypes who had high bTMB was largely reduced (12 out of 19, 63.2%) (Figure 6C). In this latter group, the number of patients with low bTMB was greatly increased (7 out of 19 patients, 36.8%) as compared to the group of patients with lost (10mfTCRVβ) clonotypes (1 of 8 patients, 12.5%) (Figure 6C), suggesting an association between lost clonotypes at 1stE and high bTMB. This association was linked to improved clinical outcomes. Thus, patients with high bTMB and disappeared 10mfTCRVβ clonotypes at 1stE had a statistically significantly better 2-year PFS compared to patients with high bTMB but without the disappearance of previously 10mfTCRVβ clonotypes from their peripheral blood (Figure 6D). We also sought to examine whether the combination of low bTMB and clonotype disappearance could correlate with PFS. However, only one patient exhibited both low bTMB and clonotype disappearance, precluding a meaningful assessment of their combined prognostic value.

## 4. Discussion

In the present study, we longitudinally analyzed the peripheral TCR β-chain repertoire of 28 patients with unresectable stage III NSCLC undergoing anti-PD-L1 immunotherapy following chemoradiotherapy. Our preliminary results demonstrate that although global TCR repertoire metrics did not significantly change between baseline and the 1stE, qualitative alterations in clonotype richness, TRBV gene usage, and dominant clonotype dynamics were associated with clinical outcomes. These findings imply that peripheral TCR repertoire remodeling reflects biologically relevant immune responses occurring during immune checkpoint blockade and may provide a useful biomarker framework for monitoring treatment efficacy.

ICIs targeting the PD-1/PD-L1 axis have revolutionized the treatment of NSCLC by restoring antitumor immunity and reinvigorating exhausted T cells within the tumor microenvironment [28,29,30]. Despite these advances, only a subset of patients experiences durable clinical benefit, underscoring the need for predictive biomarkers capable of identifying individuals most likely to respond to immunotherapy [31,32]. Since the TCR repertoire reflects the antigen-driven adaptive immune response, its characterization has emerged as a promising approach for understanding immune dynamics during ICI [33]. High-throughput sequencing technologies have enabled detailed characterization of TCR diversity and clonotype composition, providing insight into how T-cell populations respond to tumor antigens and immunotherapeutic interventions [16,34]. Previous studies have demonstrated that TCR repertoire features in both tumor tissue and peripheral blood may correlate with clinical outcomes in patients receiving ICIs [14,35]. In NSCLC specifically, the diversity of the circulating TCR repertoire prior to treatment has been associated with improved responses and longer progression-free survival following PD-1/PD-L1 blockade [36].

In our cohort, global repertoire metrics, including diversity, richness, and evenness, did not significantly differ between baseline and the first 1stE. Similar findings have been reported in other studies investigating early immunotherapy responses, where large-scale shifts in overall TCR diversity may not occur during the initial phases of treatment [37,38]. These observations suggest that early immunotherapy responses may instead be driven by more subtle clonal dynamics, including expansion or contraction of specific T-cell populations. Indeed, although cohort-wide statistical differences were not detected, patients could clearly be separated into two groups according to changes in clonotype richness at the first 1stE. One subgroup demonstrated increased clonotype counts and higher repertoire diversity, whereas the other exhibited reduced clonotype richness accompanied by decreased diversity and convergent TCR frequency. A broad and diverse TCR repertoire is thought to increase the likelihood of recognizing tumor-derived neoantigens, thereby enhancing the probability of mounting effective antitumor immune responses [33,39]. Consistent with this concept, previous studies have shown that greater TCR diversity is associated with improved outcomes in multiple cancer types treated with checkpoint inhibitor ICIs [40,41].

In addition to overall repertoire complexity, we identified differences in the usage frequencies of specific TRBV genes between patients with favorable and unfavorable clinical outcomes. In particular, higher frequencies of TRBV20-1 and lower frequencies of TRBV28 were observed among patients with improved survival and those without early disease progression. The preferential usage of certain TRBV gene segments likely reflects antigen-driven clonal selection within the T-cell repertoire [42]. Analyses of tumor-infiltrating lymphocytes have demonstrated that specific TCR Vβ families may become enriched during immune responses against tumor antigens, suggesting that V-gene usage may reflect underlying antigenic stimulation [33,43]. Interestingly, within the subgroup of long-term survivors, we observed a decline in the peripheral frequencies of TRBV15 and TRBV20-1 at the 1stE compared with baseline. One potential explanation for this observation is the redistribution of activated tumor-reactive T cells from the peripheral circulation into tumor tissues following ICI. Restoration of T-cell effector function by PD-1/PD-L1 inhibitors promotes proliferation and trafficking of tumor-specific T cells toward the tumor microenvironment [44]. Previous studies have demonstrated that immunotherapy can induce clonal replacement or redistribution of tumor-specific T cells, resulting in dynamic changes in peripheral TCR repertoires during treatment [45,46,47].

Although the present study focused on peripheral T-cell repertoire dynamics, responses to PD-L1 blockade are determined by the broader tumor immune microenvironment. In addition to T lymphocytes, innate immune cells, particularly tumor-associated macrophages (TAMs), play a critical role in regulating antitumor immunity and therapeutic responsiveness. Depending on their activation state, TAMs can either support T-cell activation or establish an immunosuppressive milieu through the production of inhibitory cytokines, modulation of antigen presentation, and promotion of T-cell exhaustion [48,49,50]. As reviewed by Roudi et al. [51], macrophage-mediated immune regulation represents a major mechanism of both primary and acquired resistance to immune checkpoint inhibitors and may substantially influence adaptive immune responses within tumors. Therefore, the TRBV repertoire alterations and clonotype dynamics observed in our study may not solely reflect direct effects of PD-L1 blockade on T cells but may also represent downstream consequences of broader immune remodeling involving innate immune populations. Furthermore, all patients in our cohort received chemoradiotherapy before durvalumab administration. Previous evidence summarized by Petrelli et al. [52]. suggests that chemotherapy can enhance antitumor immunity through increased tumor antigen release, improved antigen presentation, and modulation of suppressive immune populations, thereby potentially shaping the baseline TCR repertoire and subsequent responses to immunotherapy. Finally, the biological effects observed in our cohort should be interpreted in the context of PD-L1 blockade specifically, as anti-PD-L1 and anti-PD-1 therapies exhibit distinct mechanisms of action and may differentially affect immune-cell interactions within the tumor microenvironment, as discussed by Tartarone et al. [53]. Collectively, these observations suggest that the TCR repertoire signatures identified in our study likely reflect complex interactions between adaptive and innate immune mechanisms and support future studies integrating TCR profiling with a comprehensive characterization of the tumor immune microenvironment to better define the determinants of response and resistance to immunotherapy in NSCLC.

Another key observation of our study is that the disappearance of dominant clonotypes from peripheral blood after immunotherapy was associated with improved PFS. A subset of patients demonstrated loss of one or more of their most frequent clonotypes at 1stE, suggesting dynamic immune remodeling during treatment. Changes in highly expanded clonotypes have previously been linked to effective antitumor immune responses, reflecting antigen-driven expansion and migration of tumor-reactive T cells [54,55,56]. Moreover, analyses of tumor-infiltrating T-cell populations have shown that dominant clonotypes often represent tumor-specific lymphocytes capable of mediating cytotoxic responses [57,58,59]. Interestingly, several of the disappearing clonotypes identified in our cohort were associated with specific TRBV genes, including TRBV20-1, TRBV27, and TRBV28, further highlighting the potential relevance of these Vβ families in shaping immune responses during PD-L1 blockade. Additionally, patients exhibiting clonotype disappearance displayed distinct baseline repertoire characteristics, including higher frequencies of TRBV20-1 and TRBV7-2 and lower frequencies of TRBV21-1. These findings indicate that the baseline architecture of the TCR repertoire may influence the capacity of the immune system to undergo effective immunological remodeling during treatment.

The association of certain TRBV gene families, including TRBV20-1, TRBV27, and TRBV28, with clinical outcomes should be interpreted cautiously. TRBV gene usage reflects the variable region employed by expanding T-cell clones and may serve as an indirect marker of antigen-driven immune responses rather than possessing intrinsic biological activity. Expansion of specific TRBV families during PD-L1 blockade may therefore indicate selective proliferation of T-cell clones recognizing tumor-associated neoantigens or other immunogenic epitopes. However, the antigen specificities and functional properties of TRBV20-1-, TRBV27-, and TRBV28-expressing T cells have not been well characterized in NSCLC, and direct mechanistic evidence linking these TRBV families to checkpoint inhibitor efficacy is currently lacking. Consequently, our observations should be regarded as hypothesis-generating and warrant validation through larger studies incorporating paired TCR sequencing, antigen-specific functional assays, and independent patient cohorts.

The association between TRBV family usage and clinical outcome suggests that baseline TCR repertoire architecture may shape subsequent immune responsiveness to PD-L1 blockade. TRBV gene usage is increasingly recognized as non-random, reflecting structural constraints in antigen recognition and HLA-restricted selection pressures. Prior studies have demonstrated biased TRBV segment utilization in tumor-infiltrating lymphocytes and antigen-driven expansions, supporting the idea that TRBV skewing can reflect selection for tumor-reactive specificities [60,61,62]. In particular, TRBV20-1 and TRBV7-2 have been recurrently observed among expanded clonotypes in both tumor-associated and peripheral immune responses in cancer, suggesting their involvement in immunodominant antigen recognition programs [12,61]. In this context, the higher baseline frequency of specific TRBV families in patients exhibiting subsequent clonotype disappearance may reflect a pre-existing antigen-experienced repertoire that is predisposed for dynamic remodeling under PD-L1 blockade. Immune checkpoint inhibition can profoundly alter T-cell trafficking and clonal distribution, enabling the expansion and redistribution of tumor-reactive T cells across peripheral and tumor compartments [59,63]. We therefore hypothesize that the observed “disappearance” of dominant clonotypes from peripheral blood likely reflects immune compartmental redistribution rather than true clonal deletion. Indeed, multiple TCR sequencing studies tracking paired blood and tumor samples have shown that tumor-reactive clonotypes often decrease in peripheral blood during effective immunotherapy while simultaneously expanding within tumor tissue, consistent with trafficking of activated effector T cells into the tumor microenvironment, where they exert cytotoxic function [59,62,64]. In this model, contraction of dominant peripheral clonotypes may serve as a surrogate marker of successful immune recruitment to the tumor site, thereby explaining its association with improved progression-free survival. Alternatively, PD-L1 blockade may promote broad immune repertoire reshaping, characterized by expansion of newly primed tumor-specific clones and relative contraction of pre-existing dominant clonotypes, including those that may represent bystander responses to chronic viral or inflammatory antigens [59,63]. These mechanisms are not mutually exclusive and likely coexist during effective antitumor immune activation. Importantly, the disappearance of dominant clonotypes from peripheral blood does not necessarily imply loss of tumor specificity. Instead, it may reflect redistribution between blood and tissue compartments or competitive replacement within an evolving immune landscape driven by checkpoint blockade [62,64]. This interpretation is consistent with evidence that effective antitumor responses are associated with dynamic clonal turnover rather than static persistence of a fixed tumor-reactive repertoire.

Finally, regarding clinical applicability, TCR sequencing is currently more resource-intensive than conventional biomarkers; however, its feasibility is increasing due to reductions in sequencing costs and the development of standardized targeted TCRβ sequencing assays. Importantly, clinically actionable information may not require full repertoire reconstruction but could rely on simplified metrics such as top-clone dynamics, clonality indices, or contraction/expansion signatures derived from peripheral blood [12,60]. The minimally invasive nature of blood-based sampling enables longitudinal monitoring, which is a key advantage over tissue-based approaches. Collectively, these findings support the potential of peripheral TCR repertoire dynamics as a translationally relevant biomarker for immune checkpoint blockade, although further validation in larger independent cohorts is warranted.

We also explored the relationship between TMB and TCR repertoire characteristics. TMB has been widely investigated as a surrogate marker of tumor neoantigen load and has been associated with responsiveness to ICIs across multiple cancer types [23,65]. In NSCLC, tumors with higher mutational burdens are more likely to generate immunogenic neoantigens capable of stimulating T-cell responses [66]. Consistent with this concept, patients in our cohort with high baseline bTMB exhibited greater clonotype richness in their peripheral TCR repertoire compared with patients with low bTMB.

Furthermore, the disappearance of dominant clonotypes was enriched among patients with high bTMB, and the combination of high bTMB and clonotype loss was associated with improved PFS. These observations suggest that genomic and immunological biomarkers capture complementary aspects of tumor–immune interactions. While TMB reflects the mutational landscape and potential neoantigen repertoire of the tumor, TCR sequencing provides direct insight into the adaptive immune response generated against these antigens [67].

An additional consideration when interpreting our findings is the potential impact of prior radiotherapy on the observed immune dynamics. Beyond its direct cytotoxic effects, radiotherapy is increasingly recognized as a potent immunomodulatory intervention capable of reshaping the tumor immune microenvironment. Radiation-induced immunogenic cell death can promote tumor antigen release, enhance antigen presentation, increase T-cell priming, and facilitate recruitment of effector lymphocytes into tumor sites [68,69,70]. Furthermore, radiotherapy may influence both adaptive and innate immune compartments, including dendritic cells, macrophages, and other myeloid populations that contribute to antitumor immunity and responsiveness to immune checkpoint blockade [69,70,71]. Clinical and translational studies have suggested that these immunological effects may augment the efficacy of PD-1/PD-L1 inhibitors and contribute to the therapeutic benefit observed following chemoradiotherapy and consolidation durvalumab in unresectable stage III NSCLC [18,72,73]. Therefore, the TCR repertoire alterations observed in our study likely reflect the combined effects of prior chemoradiotherapy and subsequent PD-L1 inhibition rather than the exclusive effects of durvalumab treatment. Although this treatment sequence reflects real-world clinical practice and the PACIFIC treatment paradigm, the relative contribution of radiotherapy-induced immune remodeling to the identified TRBV signatures and clonotype dynamics cannot be determined in the present study and should be addressed in future investigations incorporating treatment-stratified analyses and longitudinal immune monitoring.

**Figure 7:**
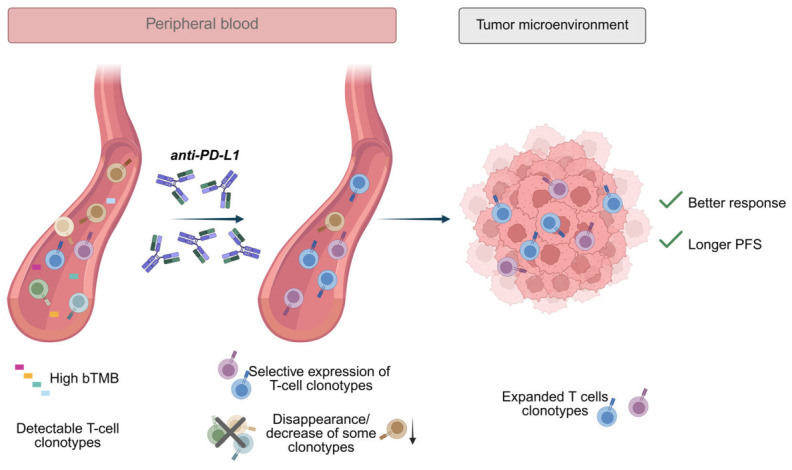
Result-generated hypothesis explaining the association of favorable patient outcomes with dominant TCR clonotype disappearance of the peripheral blood and high bTMB. Tumor-reactive T-cell clones are selectively expanded upon immunotherapy (first arrow) and migrate to the tumor tissue (second arrow) to exert their local anti-tumor effects [Created in BioRender. Fortis, S. (2026) https://BioRender.com/dp37zeb, accessed on 9 July 2026]. Agreement license VZ29WQ2VDF. bTMB, blood tumor mutational burden; PFS, progression-free survival.

Overall, the key finding of the present study is that the disappearance of TCR clonotypes from the peripheral blood of NSCLC patients after anti-PD-L1 treatment, either as a sole finding or combined with high bTMB, is associated with improved 2-year PFS. A plausible explanation for this observation is that these dominant tumor-reactive T-cell clones were selectively expanded upon immunotherapy and migrated to the tumor tissue to exert their local anti-tumor effects; however, this is only a result-generated hypothesis, since our data do not directly demonstrate T-cell trafficking into the tumor. As a result, some TCR clonotypes that were detectable in the peripheral blood before treatment may decrease or disappear from the blood because they have redistributed to tumor tissue. Their accumulation in the tumor could contribute to improved tumor control and longer PFS (Figure 7).

Despite these promising findings, several limitations of the present study should be considered. First, the relatively small sample size may limit statistical power and increase the possibility of errors. In this context, although several TRBV genes exhibited nominally significant differences between patient subgroups, only a restricted number remained significant after the Benjamini–Hochberg correction for multiple testing. Given the limited sample size and the large number of comparisons performed, these findings should be considered exploratory and interpreted with caution, while validation in an independent larger cohort is warranted. Although previous studies have reported associations between TCR repertoire diversity and clonality and response to immunotherapy, we did not observe significant differences in these metrics according to clinical outcome in our cohort. This may reflect the limited sample size and the resulting reduced statistical power, as well as differences in patient population, treatment regimens, and sequencing methodology. Second, TCR repertoire analysis was restricted to peripheral blood, which may not fully represent the immune landscape within the tumor microenvironment. Integrating analyses of tumor-infiltrating lymphocytes with peripheral blood TCR profiling may therefore provide a more comprehensive understanding of immunotherapy-induced immune dynamics. This would also shed light on whether our proposed hypothesis on the disappearance of TCR clonotypes from the peripheral blood stands; since paired blood–tumor TCR sequencing was not performed, the proposed mechanism remains speculative, and alternative explanations, including trafficking to other tissues or physiological changes in the circulating T-cell repertoire, cannot be excluded. Third, although we explored whether combining clonotype disappearance with baseline bTMB could improve prognostic stratification, the limited number of patients exhibiting both features prevented a meaningful evaluation of their combined predictive value. Larger studies are needed to determine whether longitudinal TCR repertoire changes provide prognostic information beyond established biomarkers such as bTMB. Finally, since our patients received chemoradiotherapy prior to durvalumab administration, the observed TCR repertoire changes may partially reflect radiotherapy-induced immune remodeling, making it difficult to fully distinguish the immunological effects of PD-L1 blockade from those of preceding treatment modalities. Nonetheless, the present study supports the growing evidence that peripheral TCR repertoire profiling can serve as a minimally invasive biomarker for monitoring immune responses during ICI therapy. Undoubtedly, the associations identified between specific TRBV families, clonotype dynamics, bTMB, and clinical outcomes require confirmation in larger, independent prospective cohorts before their utility as predictive biomarkers can be established.

## 5. Conclusions

Our study highlights that dynamic, qualitative changes in the peripheral TCRβ repertoire are closely linked to clinical outcomes in patients with unresectable stage III NSCLC receiving anti-PD-L1 therapy following chemoradiotherapy. Specifically, alterations in clonotype richness, biased TRBV gene usage, and the disappearance of dominant clonotypes appear to reflect biologically meaningful immune remodeling associated with effective antitumor responses. The observed interplay between TCR repertoire features and TMB further underscores the value of integrating immunological and genomic biomarkers to capture complementary dimensions of tumor–immune interactions. Although these findings should be considered exploratory and require validation in larger, independent cohorts, they suggest that peripheral TCR profiling may offer a promising, minimally invasive approach for monitoring immunotherapy-induced immune dynamics and could contribute to the future development of more refined biomarker-driven patient stratification strategies in NSCLC.

## Figures and Tables

**Table 1:** Clinicopathological and treatment characteristics of the non-small cell lung cancer (NSCLC) patients enrolled in the present study.

Characteristic	Value
Median age at diagnosis, years (range) (*n* = 28)	68.7 (48.1–80.8)
Sex	
Men	23
Women	5
Race	
Caucasian	28
Smoking status	
Active smokers	7
Former smokers	18
Non-smokers	3
Median pack-years (range)	100 (15–180)
Familial history	
Unknown	17
Yes	3
No	8
Histological type	
Squamous	15
Adenocarcinoma	12
Undifferentiated	1
Chemotherapy (CT)	
Median CT schedule, days (range)	106 (25–252)
Radiotherapy (RT)	
Complete	23
Incomplete	4
Unknown	1
Median complete RT schedule, days (range)	50.5 (32–81)
ECOG status before immunotherapy	
0	18
1	9
Unknown	1

**Table 2:** Univariate and multivariate survival analyses (Cox regression) for the evaluation of the prognostic significance of TCR clonotype disappearance in association with selected covariates.

Univariate analysis	Progression-free survival (PFS)		
	*p*	Hazard Ratio	95% CI
TCR clonotype disappearance	0.0441	4.619	1.277 to 29.56
Smoking status (smoker/non- or ex-smoker	0.3336	1.629	0.5945 to 4.463
Age	0.0487	1.079	1.003 to 1.169
ECOG (1/0)	0.7348	0.8324	0.2614 to 2.299
Histological type (squamous/adenocarcinoma)	0.0849	2.579	0.9183 to 8.329
Multivariate analysis	Progression-free survival (PFS)		
	*p*	Hazard Ratio	95% CI
TCR clonotype disappearance	0.1661	3.557	0.6471–27.86
Smoking status (smoker/non- or ex-smoker	0.6217	1.363	0.4087–5.001
Age	0.0488	1.08	1.003–1.171
ECOG (1/0)	0.5292	1.472	0.4133–4.808
Histological type (squamous/adenocarcinoma)	0.0621	3.429	1.004–14.03

## Data Availability

The datasets presented in this article are not readily available because they are part of an ongoing study. Requests to access the datasets should be directed to the corresponding authors.

## Supplementary Materials

The following supporting information can be downloaded at https://www.mdpi.com/article/10.3390/cancers18142241/s1. Figure S1: Association of clinical outcomes with major TCR repertoire metrics in the study’s patients. A. Differences in baseline vs. 1st evaluation clonotype count in patients that were alive at last follow-up (left panel) and in non-survivors (right panel). B. Differences in baseline vs. 1st evaluation clonotype Shannon diversity in patients that were alive at last follow-up (left panel) vs. non-survivors (right panel). C. Differences in baseline (left panel) and 1st evaluation (right panel) clonotype count in patients that were alive at last follow-up vs. non-survivors. D. Differences in baseline (left panel) and 1st evaluation (right panel) clonotype Shannon diversity in patients that were alive at last follow-up vs. non-survivors. For A and B, the non-parametric Wilcoxon test was used for the identification of statistically significant differences. For C and D, the non-parametric Mann–Whitney test was used. Figure S2: Identification of T-cell receptor variable β clonotypes with descending clonal frequencies in the peripheral blood of patients with non-small cell lung cancer (NSCLC) after immunotherapy. The depicted lines represent the transition of each described clonotype from the top 10 most frequent clonotypes at baseline to their ranked frequency at 1st evaluation, with numbers indicating the exact frequency rank. Red arrows indicate clonotypes that belonged to the top 10 most frequent TCRVβ clonotypes at baseline and disappeared from the peripheral blood of the corresponding patients at 1st evaluation.

## Abbreviations

The following abbreviations are used in this manuscript:

NSCLC	Non-small cell lung cancer
ICIs	Immune checkpoint inhibitors
PD-1	Programmed cell death-1
PD-L1	Programmed death-ligand 1
TAMS	Tumor-associated macrophages
TCR	T-cell receptor
TMB	Tumor mutational burden
CT	Chemotherapy
CRT	Chemoradiatiotherapy
EMA	European Medicines Agency
NCCN	National Comprehensive Cancer Network
ECOG	Eastern Cooperative Oncology Group
EDTA	Ethylenediaminetetraacetic acid
ICI	Immune checkpoint inhibition
CDR3	Complementary-determining region 3
AA	Amino acid
cfDNA	Cell-free DNA
ctDNA	Circulating tumor DNA
UMI	Unique molecular identifier
BRFAA	Biomedical Research Foundation Academy of Athens
GATK	Genome analysis toolkit
bTMB	Blood plasma tumor mutational burden
TSO500	True Sight Oncology 500
CH	Clonal hematopoiesis
VAC	Variant allele count
SD	Standard deviation
OS	Overall survival
PFS	Progression-free survival
Mb	Megabase
TRBV	TCR beta variable
CF	Clonal frequency
PD	Progressive disease
TRBJ	TCR beta joining
